# Effect of Defects and Oxidation on CNT–Copper Interface: First-Principles Calculation and Experiment

**DOI:** 10.3390/ma16216845

**Published:** 2023-10-25

**Authors:** Boyu Ju, Yubo Zhu, Wenshu Yang, Jinpeng Sun, Haozhe Li, Feng Yuan, Ziyang Xiu

**Affiliations:** 1 Harbin Institute of Technology, School of Materials Science and Engineering, Harbin 150001, China; juboyu_hit@163.com (B.J.); yws001003@163.com (W.Y.); sunjinpeng136@163.com (J.S.); lihaozhe_hit@163.com (H.L.); 2 Beijing Institute of Electronic Engineering, Beijing 100854, China; zhuyubomvp@163.com; 3 Department of Physical Education, China Agricultural University, Beijing 100083, China

**Keywords:** CNTs, Cu matrix composite, interface, the effects of carbon nanotube defects, copper surface oxide layer

## Abstract

In this paper, the effects of carbon nanotube defects and a copper surface oxide layer on a carbon nanotube–copper interface were studied via first-principles. A defect-free CNT-Cu interface, Stone–Wales defect CNT-Cu interface, single-hole and double-hole defect CNT-Cu interface, and Cu_2_O-Cu interface were simulated and calculated. By simulating the differential charge density, atomic population, bond population and density of states of the interface model, the effects of various defects on the interface bonding and electrical conductivity of the composites during the preparation of the CNT-reinforced copper matrix composites were analyzed, which provided theoretical guidance for the preparation of CNT/Cu composites. After that, copper matrix composites with different CNT defect contents were prepared via different rolling deformation processes. Their hardness and electrical conductivity were tested, and the results were consistent with the results obtained via the first-principles calculations.

## 1. Introduction

Carbon nanotubes (CNTs) are widely used in many composites, including polymer [1,2,3], metal [4,5,6] and ceramic [7,8] matrix composites, due to their excellent mechanical properties, such as their high Young ‘s modulus (more than 1 TPa) and tensile strength (more than 100 GPa) [9,10,11]. Moreover, CNTs also have extremely high thermal conductivity (3000–6000 W/m·K) and an extremely low thermal expansion coefficient (≈10^−6^ K^−1^), making CNTs an excellent nanofiller that can improve the mechanical, electrical and thermal properties of composites [12,13]. CNT-reinforced resin matrix composites have been widely studied and developed rapidly due to their good interface bonding and simple processing conditions [14,15,16]. However, there are still many challenges when CNTs are used as reinforcements in metal and ceramic matrix composites, such as difficulty in dispersion, poor interface bonding and difficult processing conditions [17,18], so their development speed is much slower.

Although the development of CNT-reinforced metal matrix composites is not yet mature [19,20], they have broad application prospects in industry due to their potential excellent mechanical properties and functional properties [21,22,23]. For example, CNT-reinforced aluminum and magnesium matrix composites can be used as structural materials in the aerospace and marine fields due to their light weight, high strength and corrosion resistance [24,25]. CNT-reinforced gold, silver and other composite materials can be used in the fields of photocatalysis, energy storage and sensors with a high surface area and high catalytic activity [26]. In addition, CNT-reinforced copper matrix composites can be used as functional materials, such as chip interconnection materials and nanosensors, with high electrical conductivity, high thermal conductivity and a high field emission efficiency [27,28,29]. Therefore, the research and application of CNT-reinforced metal matrix composites are of great significance and value.

The advantage of CNT-reinforced copper matrix composites is that they are expected to improve the hardness and strength of the Cu matrix and to leave the thermal conductivity and electrical conductivity of the Cu matrix unchanged or even greatly improve it [30]. Feng et al. [31] prepared CNT/Cu composites with good interface bonding and found that, after adding a certain content of CNTs (0.8 vol.%), the mechanical properties of the composites were greatly improved, the strength was as high as 364 MPa, and the performance was 206% higher than that of the matrix. Chen [32] prepared CNT/Cu composite wires, which have broad application prospects in the field of power transmission. Compared with the Cu matrix, the specific strength of the composite is increased by 700%, the specific conductivity is increased by 30%, and the specific ampere coefficient is increased by 110%. Chen’s study shows that CNTs are expected to simultaneously improve the mechanical and electrical properties of the Cu matrix and to greatly expand the application field of Cu materials [18]. Therefore, if CNTs are selected as the reinforcement for Cu-based composites, they will have a wider range of applications in the fields of electronics, aerospace and navigation.

The first-principles method is a reliable simulation calculation method that has been studied with respect to the properties of CNTs and Cu. In order to clarify the excellent adhesion of the Ni-SWCNTF/solder interface, Gao [33] studied the interface bonding of heterogeneous materials using density functional theory (DFT) calculations and obtained reliable results. Zhuang [34] proved via first-principles calculations that the binding energy of transition metal atoms (including Cu) with carbon nanotubes is significantly enhanced when there are vacancies in the carbon nanotubes.

In this paper, the effects of various CNT defects on a CNT-Cu interface were systematically calculated through first-principles calculations. A material preparation and performance test proved that the presence of certain defects was beneficial for improving the interface bonding of CNT-Cu and that its hardness and conductivity could be improved. 

## 2. Simulation and Experiment

### 2.1. Modeling and Methods

It is difficult to directly calculate CNTs with a large number of atoms in a complex model via first-principles calculations. In this paper, the six-membered ring structure (Gr) formed by the expansion of CNTs was used for approximate calculation, and only the single-layer structure was considered. The appropriate truncation energy E_cut_ and K-points were selected to optimize the geometry of the Gr block, and the results are shown in Table 1. The difference between the geometrically optimized Gr lattice parameters and the theoretical values was only 0.4%, indicating that the selection of optimized parameters was more reliable.

**Figure 1:**
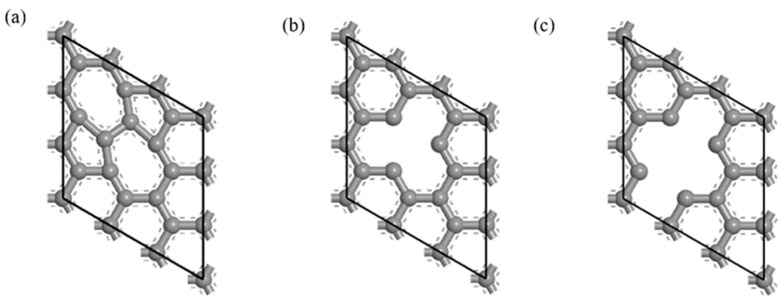
Defective Gr structure. (**a**) SW defect, (**b**) single-vacancy defect and (**c**) double-vacancy defect.

The defective CNT model was established on the optimized Gr model. The SW defect made the two adjacent six-membered ring structures in the Gr model become a five-membered ring and a seven-membered ring structure [35], as shown in Figure 1. The most common vacancy defects in CNTs include single-vacancy defects and double-vacancy defects [36], and the contents of hole defects were 5.6% and 11.1%, respectively, as shown in Figure 1.

For the optimization of the defective Gr model, the parameters were consistent with the defect-free Gr model; the Ecut was 600 eV, and the K-point was 11 × 11 × 1. The optimization results are shown in Table 2.

The Cu crystal was a face-centered cubic structure that was introduced by the crystal structure library of Materials Studio. The theoretical lattice constant is a = b = c = 3.61Å. The appropriate truncation energy Ecut and K-points were selected to optimize the geometric structure of the imported model. The results are shown in the Table 3.

Cu_2_O belongs to the three-dimensional crystal system. Each unit cell contains two oxygen atoms and four copper atoms. The oxygen atoms are arranged in a body-centered cubic arrangement, and the copper atoms are in the square gap on the side of the oxygen atoms. The theoretical lattice constant is a = b = c = 4.27Å. Through calculation, the appropriate truncation energy Ecut and K-points were selected, and the geometric optimization parameters and results of Cu_2_O bulk were finally obtained, as shown in Table 4. The difference between the geometrically optimized Cu_2_O lattice parameters and the theoretical values was only 0.7%, indicating that the selection accuracy of the optimized parameters was high.

**Figure 2:**
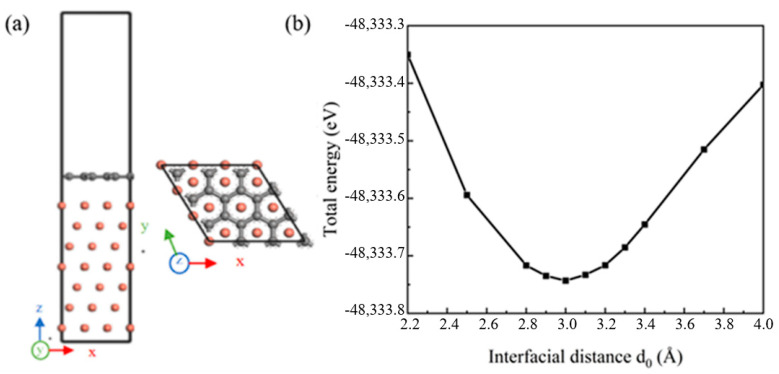
The Gr-Cu interface model and interface spacing calculation. (**a**) Interface model and (**b**) interface spacing calculation.

The defect-free single-layer Gr (001) and seven-layer Cu (111) crystal planes were selected to establish the (001) Gr/(111) Cu interface model. When the phase relationship of (001) Gr/(111) Cu interface was (100) Gr//(1$\bar{1}$0) Cu and (010) Gr//(01$\bar{1}$) Cu, the interface mismatch between Gr and Cu was 3.8%, less than 5%, which met the requirements of simulation calculation. The established interface model is shown in Figure 2a. The UBER (universal binding energy relation) test method was used to determine the appropriate interface spacing. When the interface model at a certain interface spacing had a low total energy, the interface model at that time could be considered to be the most stable. As shown in Figure 2b, the optimal interface spacing of the Gr-Cu interface model was 3.0 Å.

Then, the structure of the interface model was optimized, and the bottom four layers of Cu atoms were fixed so that the three layers of Cu atoms and Gr atoms near the interface could be fully relaxed. In order to ensure the rationality of the calculation, the DFT-D algorithm proposed by Grimme was used to correct the van der Waals force between Gr and Cu by using the pseudopotential of GGA (PBE), and the convergence quality was set to fine. Subsequent property calculations were carried out on the optimized interface model [37]. 

The interfacial adhesion work could be used to characterize the bonding properties of the interface, and its calculation formula is shown in Equation (1) [38].

(1) $$W_{ad}=\frac{E_{A}^{Slab}+E_{B}^{Slab}-E_{A/B}}{S}$$

where $W_{ad}$ is the adhesion work, $E_{A}^{Slab}$ is the total energy of A surface model after relaxation, $E_{B}^{Slab}$ is the total energy of the B surface model after relaxation, $E_{A/B}$ is the the total energy of the A–B interface model after relaxation, and S is the total energy of the A–B interface model after relaxation.

### 2.2. Experimental

#### 2.2.1. Material

The matrix used in this experiment was pure copper nanopowder. Copper nanopowder was produced by Lebo Metal Materials Technology Co., Ltd (Xingtai, China). Its morphology was spherical, with an average diameter of 600 nm. The reinforcements used were carbon nanotubes produced by Shenghui New Carbon Materials (Jiaxing, China). The purity of carbon nanotubes was more than 95%, the inner diameter was 3–5 nm, the length was 3–12 μm, and the ID/IG was 1.13.

#### 2.2.2. Preparation of Composites

CNT/Cu composites were prepared via spark plasma sintering (SPS) and vacuum hot pressing sintering (HPS). The main preparation process was divided into three stages: high-energy ball milling, sintering and directional rolling optimization. The specific parameters involved in high-energy ball milling are shown in Chapter 4, and the specific sintering parameters of SPS and HPS are shown in Chapter 5. The directional rolling parameters were as follows: rolling temperature was 800 °C, single rolling amount was 5%, and holding time between two adjacent rolling processes was 10 min.

#### 2.2.3. Characterization

Raman spectroscopy was used to characterize the lattice integrity of CNTs. Test parameters were as follows: diaphragm of 600, test laser wavelength of 532 nm and test range of 500~3000 cm^−1^. The damage degree of CNTs during ball milling could be characterized by the peak intensity ratio (I_D_/I_G_) of D peak and G peak in Raman spectroscopy. The larger the ratio, the higher the defect degree of CNTs. The conductivity of the composite material was tested using the FD-102 eddy current conductivity meter manufactured by FIRST company. The test temperature was 25 °C, and the unit was IACS%. HBV-30A Brinell hardness tester was used to test the hardness of the composite material, and unit was HBW.

## 3. Results and Discussion

**Figure 3:**
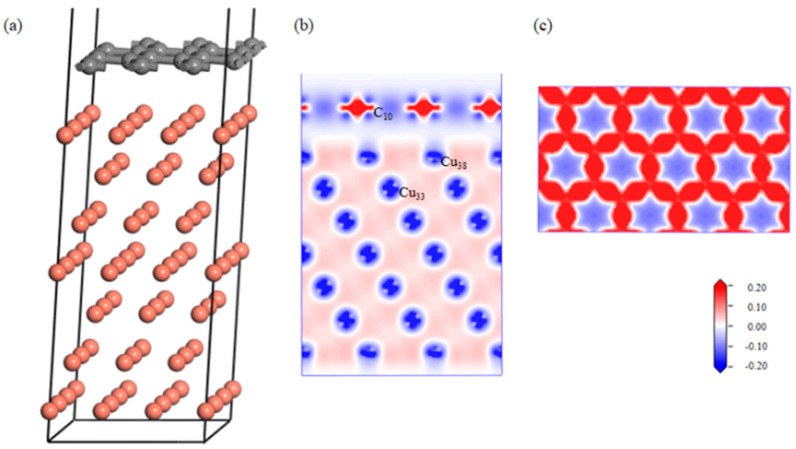
The defect-free Gr-Cu interface model and charge differential density diagram. (**a**) Optimized Gr-Cu interface model, (**b**) charge difference density diagram and (**c**) charge density diagram of Gr layer.

The effect of the defects on the graphene–aluminum interface was analyzed via first-principles calculations. After geometric optimization, the defect-free Gr-Cu interface structure was relatively flat, and the C atom and Cu atom were basically kept in their original position without a large position offset, and Figure 3 shows the differential charge density diagrams of the bounded Gr-Cu surface model and its Gr layer. It can be seen that there was no charge transfer between the C atoms and Cu atoms, there was no interaction between C and Cu, and the C-C bond of the Gr layer was not destroyed.

The bond population of the defect-free Gr-Cu interface model was analyzed, as shown in Table 5. The C-C bond length in Gr was about 1.45 Å, and the Cu-Cu bond length in Cu was about 2.53 Å. In the absence of defects, the C-Cu bond was not generated in the interface model, and interface bonding depended on the van der Waals force between the atoms.

**Figure 4:**
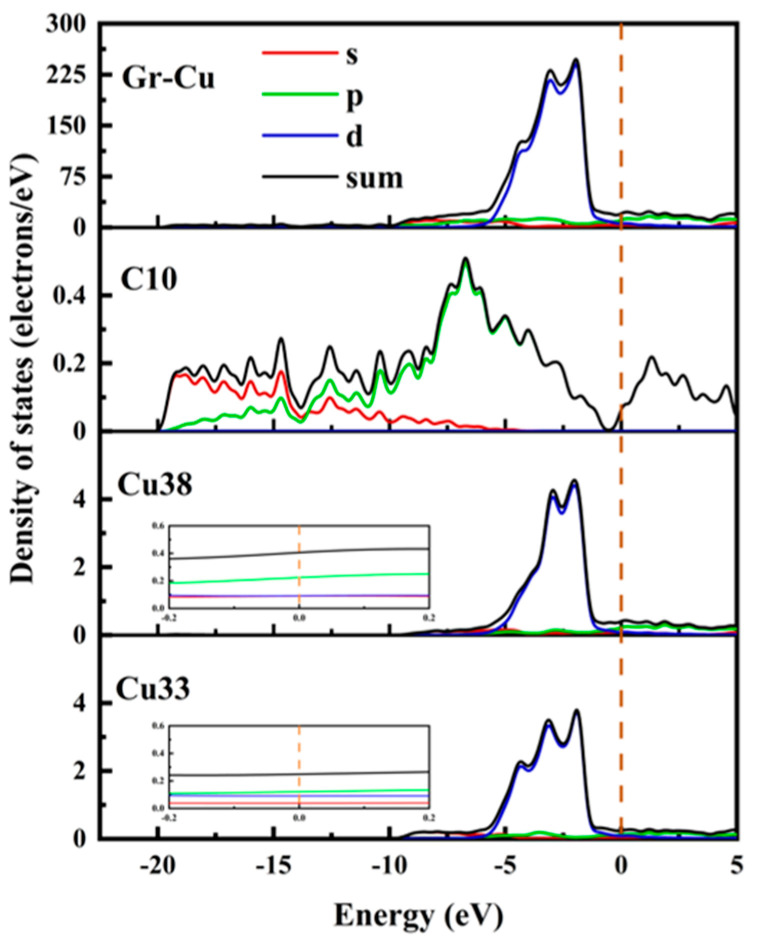
Total density of states and partial density of states of Gr-Cu interface.

The partial densities of states of the C10 atom in Gr, the Cu38 atom in the Gr-Cu interface layer and the Cu33 atom in the Cu matrix were analyzed, as shown in Figure 4. The C10 atom exhibited a very small peak at the Fermi level, which was provided by the p-orbital. This means that, when Gr is adsorbed on the Cu surface, the Cu atom will interfere with the delocalized π electrons of Gr, making Gr exhibit a certain metallicity. The value of the Cu atom at the Fermi level was not zero, showing the inherent properties of metal elements. At the Fermi level, electrons were mainly provided by the 2p orbital of C and the 2p orbital of Cu, and the electrons contributed by the Cu atoms accounted for a relatively high proportion. Compared with Cu33, the value of Cu38 at the Fermi level was increased by about 30%, indicating that Gr had a certain effect on the p-electron of the Cu atom, which made it show stronger metallicity. The electronic density of states of the C atom was more widely distributed; additionally, the electronic density of states of the Cu was is more concentrated, and the value was relatively higher.

**Figure 5:**
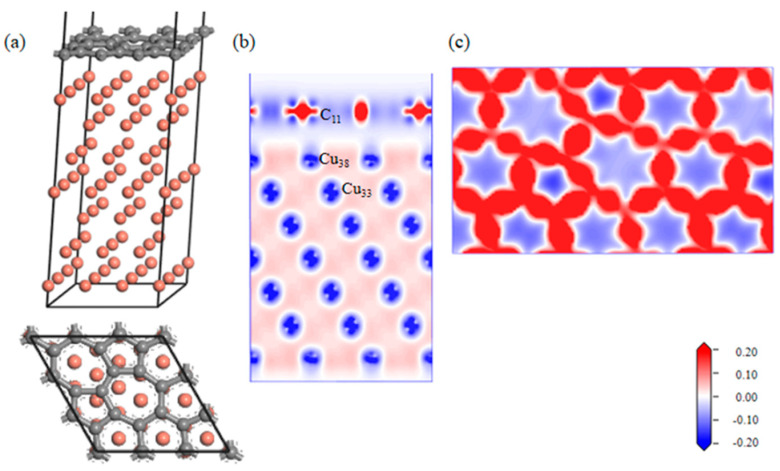
The SW defect Gr-Cu interface model and charge difference density diagram. (**a**) Optimized SW defect Gr-Cu interface model, (**b**) charge difference density diagram and (**c**) charge density diagram of Gr layer.

A geometrically optimized SW defect Gr-Cu interface model was established, as shown in Figure 5. The introduction of the SW defect made the Gr layer in the interface model distort in the direction of the Cu atoms; however, the degree of change was very small, indicating that there was a certain force between the Cu atoms and C atoms in the geometric optimization process, but it did not reach the bonding level. From the charge difference density diagram, it can be seen that there was no charge transfer between the C and Cu atoms and that there was no bonding between the C and Cu atoms, showing the same results as the defect-free Gr-Cu interface model. From the charge density diagram of the Gr layer, it can be seen that there were two seven-membered rings and two five-membered rings at the defects of the Gr layer and that the C-C bond structure was not destroyed, but the bond length was changed, showing a typical SW defect structure. The interfacial adhesion work of the SW defect Gr-Cu interface model was calculated to be 1.396 J/m^2^, which was slightly smaller than that of the defect-free Gr-Cu interface, indicating that the SW defect CNT-Cu interface was less stable and difficult to form.

The bond populations of the Gr-Cu interface model with SW defects were analyzed, as shown in Table 6. The bond length of C11-C12 in Gr was still 1.45 Å, which was the same as that of the interface model without defects. The bond length of C10-C15 was changed, and the bond length of Cu-Cu in Cu was basically unchanged. In the case of the SW defects, there was no bond between C and Cu in the interface model, and an antibond was formed between C11 and Cu38, which was not conducive to interface bonding between Gr and Cu. Therefore, bonding at the SW defect Gr-Cu interface was still physical bonding.

**Figure 6:**
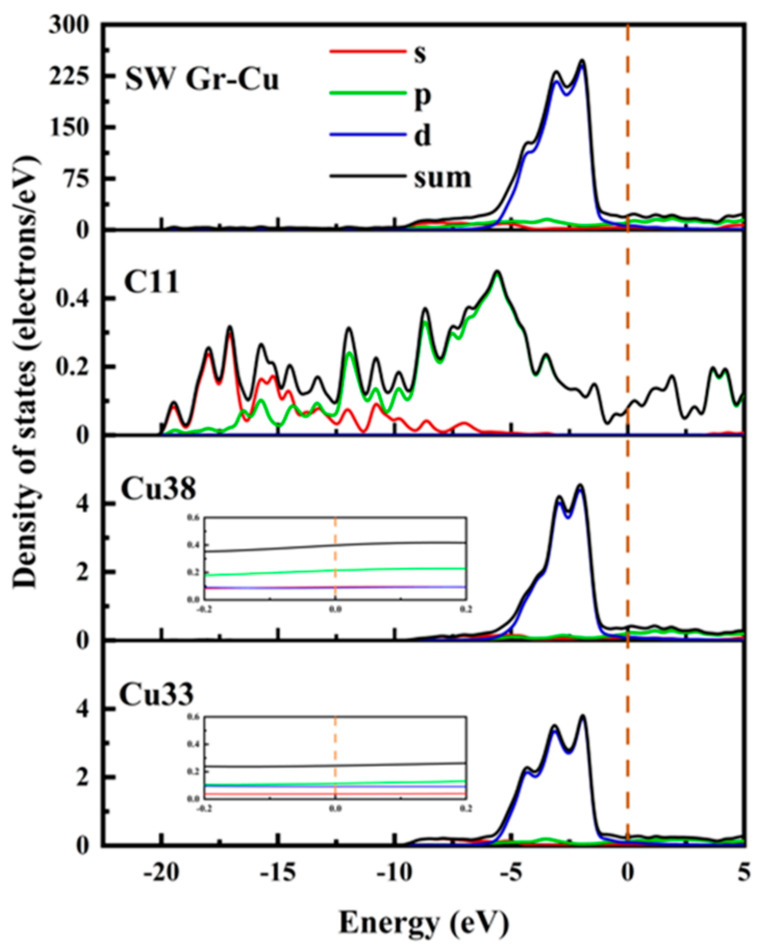
The total density of states and partial density of states of Gr-Cu interface with SW defects.

The electronic state at the Gr-Cu interface model with SW defects is shown in Figure 6. Compared with the defect-free Gr, the electronic densities of states of the Cu atoms near the SW defect and the Cu atoms inside the interface did not change significantly, which, again, indicated that the SW defect formed by the Gr layer did not have a large effect on the Cu atoms. The value of the C atom at the Fermi level at the SW defect was slightly increased, which was still mainly provided by the p orbital, indicating that the Gr at the SW defect presented a certain metallicity. The p-electron value of C increased significantly at −8.66 eV, while Cu did not change in this range, indicating that this was due to the change in the Gr model structure caused by the SW defects. The density of states diagram, again, proves that the SW defect had little effect on the Gr-Cu interface.

**Figure 7:**
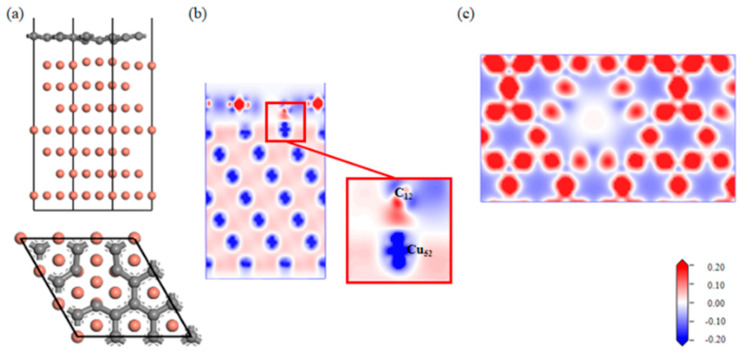
The Gr-Cu interface model with single-hole defect and charge differential density diagram. (**a**) Optimized single-hole defect Gr-Cu interface model, (**b**) charge difference density diagram and (**c**) charge density diagram of Gr layer.

A Gr-Cu interface model with a single-hole defect was established, as shown in Figure 7. The Cu atoms and C atoms near the single hole attracted each other; the C atoms decreased, and the Cu atoms increased. From the charge difference density map, it can be seen that there was a charge transfer between the C and Cu atoms, forming a covalent bond. The connection between the single-hole defect Gr-Cu interface was a mixture of physical and chemical effects, which was different from that of the defect-free Gr-Cu interface model, where chemical effects dominated. From the charge density diagram of the Gr layer, it can be seen that there was no charge transfer region at the Gr layer defect, indicating that the C-C bond structure was destroyed and that the charge transfer of the surrounding C atoms changed, showing a typical single-vacancy defect structure. The interfacial adhesion work of the single-hole defect Gr-Cu interface model was calculated to be 2.807 J/m^2^, which was 1.94 times that of the defect-free Gr-Cu interface. The magnitude of the interfacial adhesion work reflects the strength of interfacial bonding. Therefore, the introduction of the single-vacancy defects improved the bonding strength of the CNT-Cu interface.

Combined with the atomic population (Table 7) and bond population (Table 8) of the single-hole defect Gr-Cu interface, it can be seen that C12, near the single hole, was 0.18e-, Cu52 lost 0.14e-, the C12-Cu15 chemical bond length was 2.05 Å, the average bond population was 0.41, and a polar covalent bond was formed between C12 and Cu15. The introduction of a single hole made the C atoms and Cu atoms form three pairs of chemical bonds at the interface.

**Figure 8:**
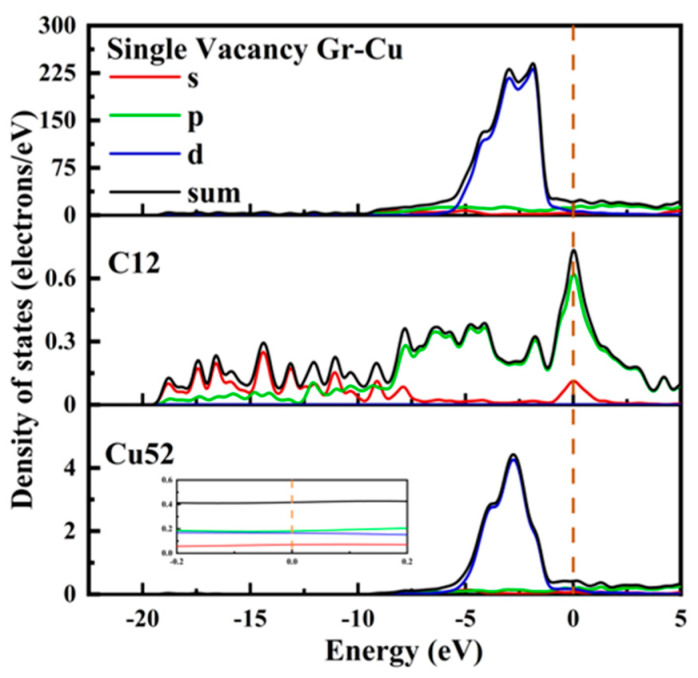
The total density of states and partial density of states of Gr-Cu interface with single-vacancy defects.

The electronic state at the Gr-Cu interface of the single-vacancy defect is shown in Figure 8. Similar to the defect-free case, the value at the Fermi level of the Cu52 atom at the interface did not change significantly and still showed the metallicity of Cu itself. In the range of 0~−5 eV, the density of states peak became sharper than that of the defect-free case, gradually changing from double peaks to single peaks. The value of the C12 atom at the Fermi level was greatly improved from semimetallic to metallic. The d-orbital electrons of Cu52 decreased in the range of 0~−5 eV, while new s-orbital and p-orbital electron peaks appeared in C12 at −4.1 eV, −1.7 eV and −0 eV, indicating that the C atoms and s- and p-orbitals near the single-hole defects were hybridized with the d-orbitals of the Cu atoms, resulting in a chemical bond between C and Cu. In addition, after the introduction of the monovacancy defects, it can be observed that the peak value of the total density of states at the Gr-Cu interface at −1.86 eV was reduced, which may adversely affect the electrical conductivity at the interface.

**Figure 9:**
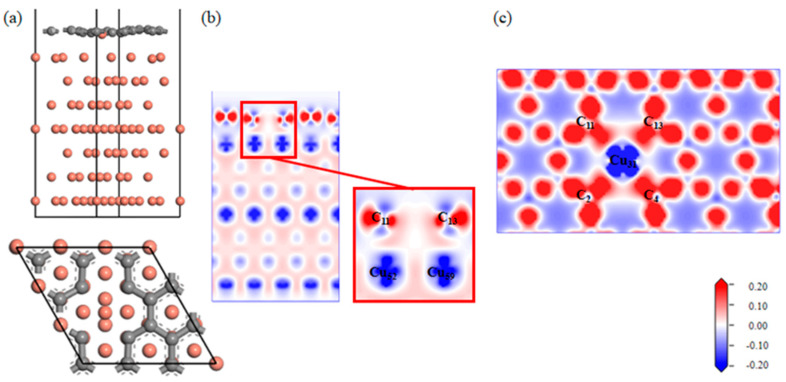
The Gr-Cu interface model with divacancy defects and charge differential density diagram. (**a**) The optimized Gr-Cu interface model with double-vacancy defects, (**b**) charge difference density diagram and (**c**) charge density diagram of Gr layer.

A Gr-Cu interface model with divacancy defects was established, as shown in Figure 9. Compared with the single-vacancy defect model, the divacancy defect had a larger defect area, which made the Cu31 at the defect enter the Gr defect. In addition, the remaining Cu atoms at the defect position still had a strong force on the C atom, thus forming a structure in which Cu was adsorbed on the Gr surface and bulged into the interface and in which the Cu atoms in the first and second layers were shifted. It can be seen from the charge difference density diagram that a charge transfer occurred between the Cu31 embedded in Gr and the C2, C4, C11 and C13 atoms at the defects, forming covalent bonds. A charge transfer occurred between Cu52 and Cu59 and C11 and C13, respectively, forming covalent bonds. At this time, there were two kinds of C-Cu bonds in the double-hole Gr-Cu interface, which were the C-Cu bonds in the Gr layer and the C-Cu bonds between the interfaces. From the charge density diagram of the Gr layer, it can be seen that the charge transfer region at the Gr layer defect was completely different from that of the single-vacancy defect. At this time, the C-C bond structure at the defect was destroyed to form a C-Cu bond structure, and the charge transfer of the surrounding C atoms changed, showing a typical double-vacancy defect structure.

In Table 9 and Table 10, it can be seen that the covalent bond formed between the Cu31 embedded in the defect and the nearby C atoms was strong, the bond population was 0.41~0.42, and the bond length was 1.94 Å~1.96 Å; the population of C11-Cu52 and C13-Cu59 was 0.18, and the bond length was 2.17 Å. The population and bond length reflect the strength of the covalent bond. At this time, the bond strength was much lower than the covalent bond between the interfaces formed by single-vacancy defects. The calculated interfacial adhesion work of the double-hole defect Gr-Cu interface model was 1.925 J/m^2^, which was 1.33 times that of the defect-free Gr-Cu interface, which was lower than that of the single-hole defect Gr-Cu interface model. The double-vacancy defect improved the interfacial bonding strength, but the C-Cu bond between the interfaces was weaker than that of the single-vacancy defect Gr-Cu interface.

**Figure 10:**
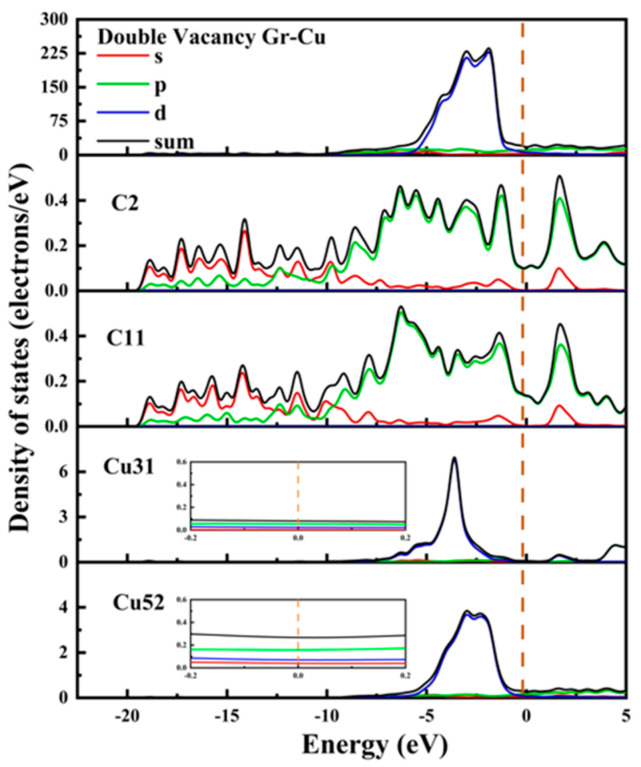
The total density of states and partial density of states of the Gr-Cu interface model with divacancy defects.

The electronic state of the Gr-Cu interface model with divacancy defects is shown in Figure 10. The value of the Cu31 embedded in the defect at the Fermi level was reduced, and its metallicity was weakened. Similar to the case of the monovacancy defects, the value at the Fermi level of the Cu52 atom at the interface did not change significantly, still showing the metallicity of Cu itself. The values of the C2 atom and C11 atom at the Fermi level were greater than zero, showing a certain metallicity. The s-orbital and p-orbital of the C2 atom and the d-orbital of the Cu31 atom were hybridized in the range of 0~−5 eV, forming a strong chemical combination. In the range of 0~−5 eV, the number of C11 and Cu52 orbital bindings decreased significantly, indicating that the chemical binding between C11 and Cu52 was weak. Due to the decrease in the number of electrons provided by the Cu31 atom at the Fermi level and the significant decrease in the peak values of the total Fermi level at the interface at −2.96 eV and −1.98, it can be considered that the divacancy defects also had an adverse effect on the electrical conductivity at the interface and that the degree of influence was greater than that of the monovacancy defects. This was mainly due to the introduction of defects that destroyed the intrinsic electrical conductivity of the Gr layer. Therefore, on the one hand, the introduction of hole defects can improve the interfacial bonding strength of CNT/Cu composites, but on the other hand, it will also reduce the conductivity of the material.

In the preparation process of CNT/Cu composites, ball milling and subsequent sintering often lead to oxidation on the surface of the Cu powder. This section studies the interface properties between Cu_2_O and CNTs so as to provide theoretical guidance for the preparation of CNT/Cu composites. Different from the interface between Cu and CNTs, the interface between Cu_2_O and CNTs had two cases of Cu cutoff and O cutoff. In this section, the two cases are compared and discussed. The CNTs were still calculated using the Gr model.

**Figure 11:**
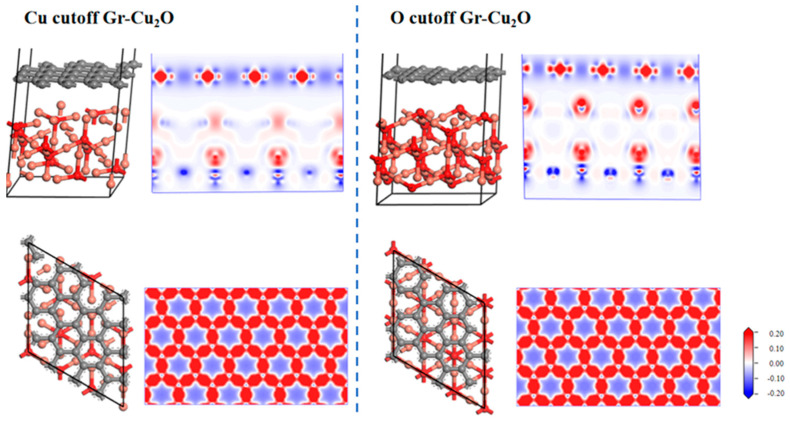
Two types of Gr-Cu_2_O interface models and charge differential density.

Figure 11 shows the two interface models after geometric optimization and their charge difference densities. It can be seen that there was no charge transfer at the interface in both cases, indicating that the interface bonding between Cu_2_O and the CNTs was still dominated by physical bonding. It can also be seen from the atomic and bond populations at the interface in Table 11 and Table 12 that there were only C-C and O-Cu bonds in the two interface models, where the C-C bond was a nonpolar covalent bond and where the O-Cu bond was a polar covalent bond. In both cases, the bond length and bond population of the C-C bond were exactly the same, both of which were 1.46Å and 1.07, while the O-Cu bond was different. In the case of the O cutoff, the bond length was shorter, and the bond population was larger and more stable. After calculation, in the case of the Cu cutoff and O cutoff, the interfacial adhesion works of the Cu_2_O-Gr interface model were 0.851 J/m^2^ and 0.637 J/m^2^, respectively, which were less than the interfacial adhesion work of the Cu-Gr interface model.

**Figure 12:**
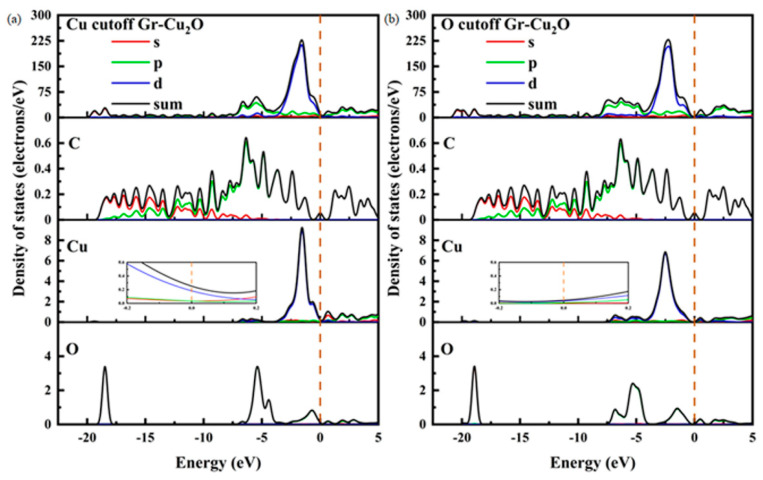
The densities of states and differential densities of states of two types of Gr-Cu_2_O interfaces. (**a**) Cu cutoff and (**b**) O cutoff.

Figure 12 shows the densities of states and the differential densities of states of different types of Gr-Cu_2_O interfaces. In both cases, the value of the interface at the Fermi level was close to zero, and compared with Gr-Cu, the density of states in the whole energy range was significantly reduced. This was due to the fact that Cu_2_O is a semiconductor. Therefore, once the Cu powder is oxidized, the oxide layer on its surface will inevitably have an adverse effect on the conductivity of the material. There was no orbital overlap between C and Cu or between C and O in the two interface models within 0~−5 eV, which, again, shows that there was no electron transfer or chemical bond formation at the Cu_2_O-Gr interface. The Cu_2_O-Gr interface was physically bonded.

**Figure 13:**
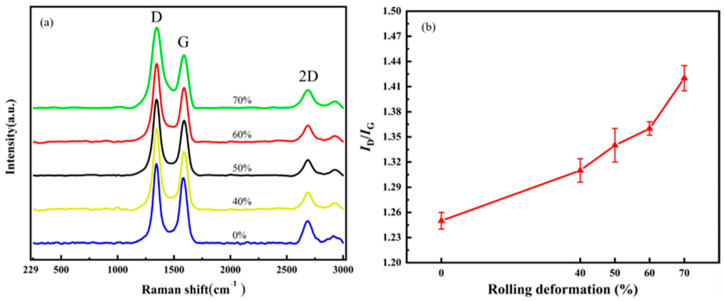
The effect of rolling reduction on the structural integrity of CNTs. (**a**) Raman spectrum and (**b**) changes in I_D_/I_G_.

CNT/Cu composites with different defect contents were prepared by adjusting the rolling deformation of the composites. The rolling deformation was 40%, 50%, 60% and 70%, respectively. Figure 13 characterizes the damage of the CNTs in the composites under different rolling deformations. Figure 13a is the Raman spectrum comparison diagram, and Figure 13b is the change diagram of I_D_/I_G_. I_D_/I_G_ = 1.25 for the CNTs in the composites without rolling; when the rolling deformation was 40%, I_D_/I_G_ = 1.31; when the rolling deformation was 50%, I_D_/I_G_ = 1.34; when the rolling deformation was 60%, I_D_/I_G_ = 1.36; and when the rolling deformation was 70%, I_D_/I_G_ = 1.42. With an increase in the rolling deformation, the value of I_D_/I_G_ also increases gradually.

**Figure 14:**
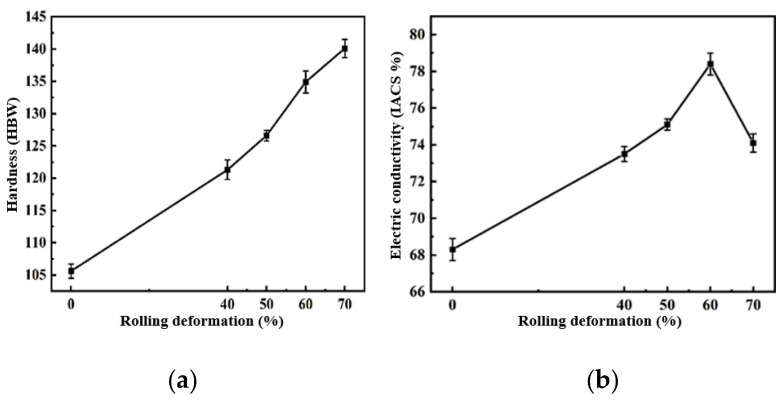
2 wt.% CNT/Cu composites with different rolling deformations. (**a**) Hardness and (**b**) conductivity.

The hardness and electrical conductivity of the composites under different rolling deformations were tested. The hardness and electrical conductivity of the 2 wt.% CNT/Cu composites changed with the rolling deformation as shown in Figure 14. This can be used to reflect the change in the behavior of the hardness and conductivity with I_D_/I_G_.

It can be seen in the figure that the hardness of the composites increased with an increase in the rolling deformation. Rolling led to an increase in I_D_/I_G_, and the hardness of the composites increased by 34.5% from 105.6 HBW to 140.1 HBW. Combined with the previous first-principles calculations, the defects of the CNTs caused by rolling were increased, and the formed single-atom defects and double-atom defects were beneficial to the chemical bonding of C and Cu at the interface, which improved the interface bonding strength and enhanced the strengthening efficiency of the CNTs. The electrical conductivity of the 2 wt.% CNT/Cu composites with the change in the rolling deformation is shown in Figure 14. The electrical conductivity of the composites increased first and then decreased with an increase in the rolling deformation. This was because the conductivity of CNT/Cu composites is affected by many factors. It is generally believed that the interface bonding mode and interface area of the material as well as the degree of dispersion and the integrity of the CNTs are the main factors affecting the conductivity of CNT/Cu composites. At the beginning of the increase in the rolling deformation, the conductivity of the composites increased by 14.8% from 68.3 IACS% to 78.4 IACS%. This was mainly because an increase in the amount of deformation improves the interface bonding of the composite material, which is conducive to the transfer of electrons and phonons. However, too many defects will cause the transfer process to act as an obstacle, making the scattering behavior worse, reducing the transfer speed and thus reducing the conductivity.

## 4. Conclusions

In this paper, the Gr-Cu interface models without defects, SW defects, single-vacancy and double-vacancy point defects were constructed by using the first-principles approximation calculation of the expanded plane structure the Gr of the CNTs, and the effects of the defects in the CNTs on the interface properties were studied. The Gr-Cu_2_O interface model under a Cu cutoff and O cutoff was constructed, and the effect of Cu powder surface oxidation on the interface of the composite was studied. The relationship between the CNT defects and strengthening behavior was verified by the experiments. The main conclusions are as follows:

(1) SW defects have little effect on the atoms at the interface. There is no electron transfer between the C atoms and O atoms, and the interface bonding is still physical bonding. The interfacial adhesion work of the SW defect Gr-Cu interface model was 1.396 J/m^2^, which was slightly smaller than that of the defect-free Gr-Cu interface (1.447 J/m^2^). The SW defect CNT-Cu interface had poor stability and was not easy to form.

(2) A single-hole defect causes a charge transfer between the Cu atom at the interface and the C atom at the defect, forming a Gr-Cu interface dominated by chemical bonding. The interfacial adhesion work of the single-hole defect Gr-Cu interface model was 2.807 J/m^2^, which was 1.94 times that of the defect-free Gr-Cu interface. The introduction of single-hole defects improved the bonding strength of the CNT-Cu interface. After the introduction of the single-hole defects, the peak of the total density of states at the Gr-Cu interface decreased at −1.86 eV, which may have had an adverse effect on the conductivity at the interface.

(3) A double-hole defect makes the Cu atoms directly opposite to the defect embed into the Gr defect, and the Cu atoms embedded in the defect form a chemical bond with the surrounding C atoms with a high bonding strength. However, since the covalent bond is located in the Gr layer at this time, the improvement in the interface strength is limited. The two Cu atoms and C atoms around the defect form a covalent bond between the interfaces, but the bond strength is much lower than that of the interface formed by a single-hole defect. The interfacial adhesion work of the double-hole defect Gr-Cu interface model was 1.925 J/m^2^, which was 1.33 times that of the defect-free Gr-Cu interface. The double-hole defect improved the interfacial bonding strength, but it was lower than that of the single-hole defect Gr-Cu interface. Compared with the single-hole defect, the double-hole defect further reduced the density of states of the Gr-Cu interface in the energy range near the Fermi level. Therefore, a hole defect will reduce the conductivity of the composite material.

(4) There is no charge transfer at Cu-cutoff and O-cutoff Gr-Cu_2_O interfaces, and the interface bonding between the two is physical bonding. In the case of a Cu cutoff and O cutoff, the interfacial adhesion works of the Cu_2_O-Gr interface model were 0.851 J/m^2^ and 0.637 J/m^2^, respectively, which were less than that of the Cu-Gr interface model. It can be seen from the density of states that Cu_2_O is a semiconductor and that its conductivity is weaker than that of metal. The oxidation of copper powder will reduce the conductivity of the composite. Therefore, the oxidation of Cu powder should be avoided in the subsequent preparation of composite materials.

In the future, we hope to improve the interface of CNTs/Cu through interfacial modification and to increase the bonding strength of CNTs and Cu by introducing an intermediate transition layer on the surface of the CNTs and Cu. The interfacial bonding of CNT-Cu under the control of the transition layer was simulated through first-principles calculations, such as the calculation of the adhesion work, layout number, interfacial bonding strength and other parameters, to screen the appropriate bonding interface, and the preparation of CNT/Cu composites modified by a transition layer was realized by the experimental design.

## Figures and Tables

**Table 1:** Geometric optimization parameters and results of Gr model.

E_cut_ (eV)	K-Point	Scf (eV/atom)	E_tol_ (eV)	a (Å)	Experiment (Å)
600	11 × 11 × 1	1.0 × 10^−6^	−315.71	2.47	2.46

**Table 2:** Geometric optimization parameters and results of defective Gr model.

Type	E_cut_ (eV)	K-Point	Scf (eV/atom)	E_tol_ (eV)	a (Å)
SW Vacancy	600	11 × 11 × 1	1.0 × 10^−6^	−2835.20	7.27
Single Vacancy				−2673.21	7.37
Double Vacancy				−2476.43	7.35

**Table 3:** Geometric optimization parameters and results of Cu model.

E_cut_ (eV)	K-point	Scf (eV/atom)	E_tol_ (eV)	a (Å)	Experiment (Å)
480	11 × 11 × 11	1.0 × 10^−6^	−6778.40	3.63	3.61

**Table 4:** Geometric optimization parameters and results of Cu_2_O model.

E_cut_ (eV)	K-point	Scf (eV/atom)	E_tol_ (eV)	a (Å)	Experiment (Å)
450	7 × 7 × 7	1.0 × 10^−6^	−1680.90	4.30	4.27

**Table 5:** Bond population analysis of defect-free Gr-Cu interface.

Interface	Bond	Population	Length (Å)
Gr-Cu	C10-C15	1.10	1.45
	C12-C17	1.10	1.45
	Cu38-Cu40	0.17	2.53
	Cu33-Cu59	0.17	2.53

**Table 6:** Geometric optimization parameters and results of the SW defect Gr-Cu interface.

Interface	Bond	Population	Length (Å)
SW defects	C11-C12	1.19	1.45
	C10-C15	1.19	1.31
	Cu38-Cu40	0.19	2.53
	Cu33-Cu59	0.18	2.54
	C11-Cu38	−0.11	3.0

**Table 7:** The atomic population of single hole defect Gr-Cu interface.

Interface	Atom	s	p	d	Total	Charge (e-)
Single-Vacancy Defect	C7	1.26	2.92	0.00	4.18	−0.18
	C12	1.26	2.92	0.00	4.18	−0.18
	C14	1.26	2.92	0.00	4.18	−0.18
	Cu31	0.54	0.63	9.69	10.86	0.14
	Cu52	0.54	0.63	9.69	10.86	0.14
	Cu59	0.54	0.63	9.69	10.86	0.14

**Table 8:** The bond population of single hole defect Gr-Cu interface.

Interface	Bond	Population	Length (Å)
Single-Vacancy Gr-Cu	C12-Cu52	0.41	2.05
	C14-Cu59	0.41	2.06
	C7-Cu31	0.41	2.06

**Table 9:** The atomic population of the Gr-Cu interface with double hole defects.

Interface	Atom	s	p	d	Total	Charge (e-)
Divacancy Defects	C2	1.26	3.07	0.00	4.33	−0.33
	C4	1.26	3.07	0.00	4.33	−0.33
	C11	1.26	3.09	0.00	4.35	−0.35
	C13	1.26	3.09	0.00	4.35	−0.35
	Cu31	0.53	0.20	9.48	10.21	0.79
	Cu52	0.54	0.63	9.73	10.9	0.10
	Cu59	0.54	0.63	9.73	10.9	0.10

**Table 10:** The bond population of Gr-Cu interface with double hole defects.

Interface	Bond	Population	Length (Å)
Divacancy Defects	C2-Cu31	0.42	1.94
	C4-Cu31	0.42	1.94
	C11-Cu31	0.41	1.96
	C13-Cu31	0.41	1.96
	C11-Cu52	0.18	2.17
	C13-Cu59	0.18	2.17

**Table 11:** The atomic population of different types of Gr-Cu_2_O interface.

Interface	Atom	s	p	d	Total	Charge (e-)
Cu-cutoff Gr-Cu_2_O	C29	1.09	2.93	0.00	4.02	−0.02
	C30	1.09	2.93	0.00	4.02	−0.02
	O10	1.84	4.80	0.00	6.64	−0.64
	Cu34	0.49	0.35	9.64	10.48	0.52
	C19	1.09	2.93	0.00	4.02	−0.02
O-cutoff Gr-Cu_2_O	C20	1.09	2.92	0.00	4.01	−0.01
	O1	1.81	4.89	0.00	6.70	−0.70
	Cu11	0.67	0.36	9.62	10.65	0.35

**Table 12:** The bond populations of different types of Gr-Cu_2_O interfaces.

Interface	Bond	Population	Length (Å)
Cu-cutoff Gr-Cu_2_O	C29-C30	1.07	1.46
	O10-Cu34	0.43	1.86
O-cutoff Gr-Cu_2_O	C19-C20	1.07	1.46
	O1-Cu11	0.46	1.80

## Data Availability

The data presented in this study are available on request from the corresponding author.
